# Development of therapeutic vaccines for the treatment of diseases

**DOI:** 10.1186/s43556-022-00098-9

**Published:** 2022-12-08

**Authors:** Yaomei Tian, Die Hu, Yuhua Li, Li Yang

**Affiliations:** 1 College of Bioengineering, Sichuan University of Science & Engineering, No. 519, Huixing Road, Zigong, Sichuan 643000 The People’s Republic of China; 2 State Key Laboratory of Biotherapy and Cancer Center/Collaborative Innovation Center for Biotherapy, West China Hospital, Sichuan University, Chengdu, 610041, China No. 17, Section 3, South Renmin Road, Chengdu, Sichuan 610041 The People’s Republic of China; 3 Department of Arboviral Vaccine, National Institutes for Food and Drug Control, Tiantan Xili, Dongcheng District, Beijing, 100050 The People’s Republic of China

## Abstract

Vaccines are one of the most effective medical interventions to combat newly emerging and re-emerging diseases. Prophylactic vaccines against rabies, measles, etc., have excellent effectiveness in preventing viral infection and associated diseases. However, the host immune response is unable to inhibit virus replication or eradicate established diseases in most infected people. Therapeutic vaccines, expressing specific endogenous or exogenous antigens, mainly induce or boost cell-mediated immunity via provoking cytotoxic T cells or elicit humoral immunity via activating B cells to produce specific antibodies. The ultimate aim of a therapeutic vaccine is to reshape the host immunity for eradicating a disease and establishing lasting memory. Therefore, therapeutic vaccines have been developed for the treatment of some infectious diseases and chronic noncommunicable diseases. Various technological strategies have been implemented for the development of therapeutic vaccines, including molecular-based vaccines (peptide/protein, DNA and mRNA vaccines), vector-based vaccines (bacterial vector vaccines, viral vector vaccines and yeast-based vaccines) and cell-based vaccines (dendritic cell vaccines and genetically modified cell vaccines) as well as combinatorial approaches. This review mainly summarizes therapeutic vaccine-induced immunity and describes the development and status of multiple types of therapeutic vaccines against infectious diseases, such as those caused by HPV, HBV, HIV, HCV, and SARS-CoV-2, and chronic noncommunicable diseases, including cancer, hypertension, Alzheimer’s disease, amyotrophic lateral sclerosis, diabetes, and dyslipidemia, that have been evaluated in recent preclinical and clinical studies.

## Introduction

**Fig. 1:**
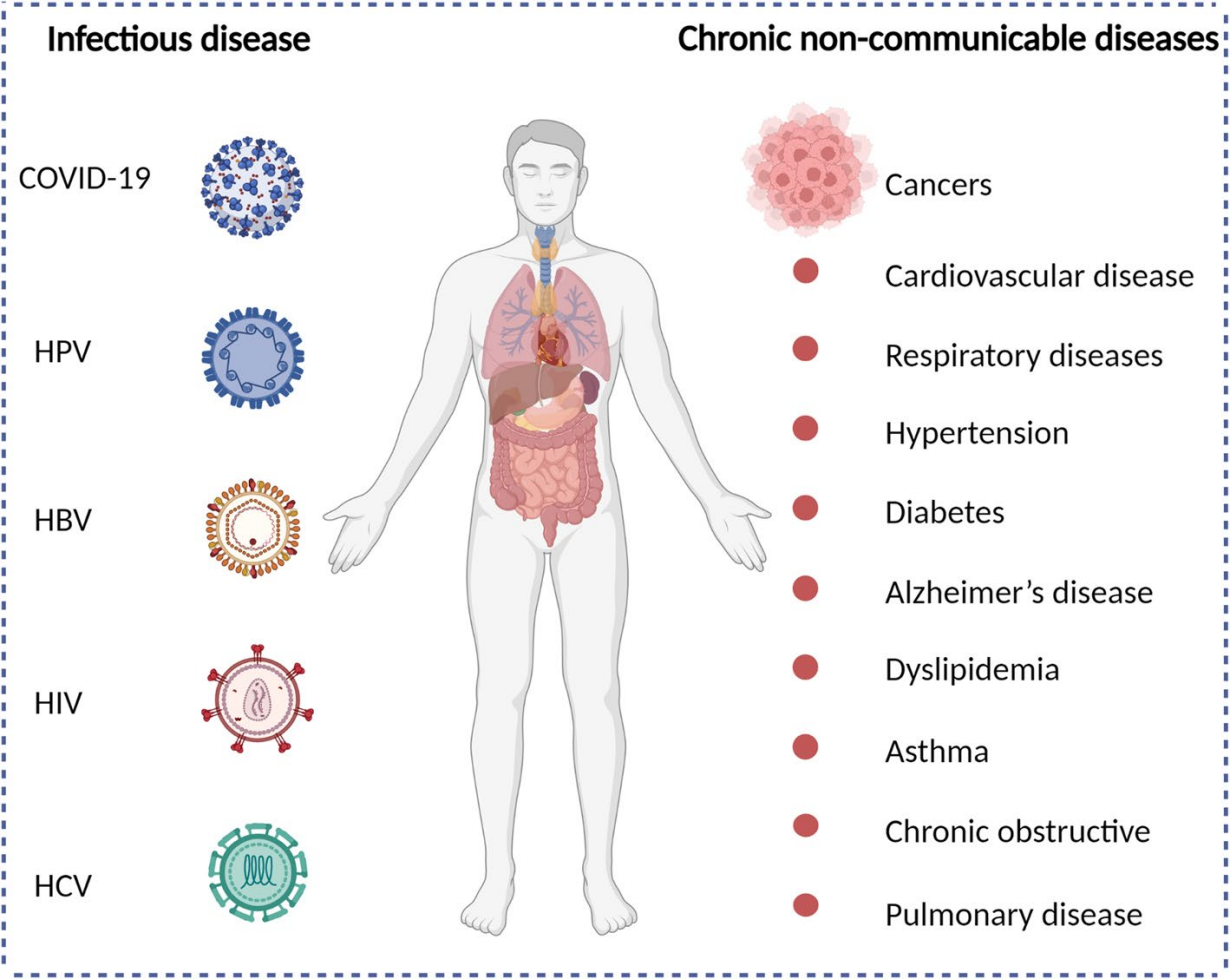
A summary of infectious disease and chronic non-communicable diseases

Infectious diseases are previously the leading cause of death [1].Vaccines have traditionally been used as the most effective medical interventions to reduce the death and morbidity caused by infectious diseases [2]. Vaccination has significantly reduced the burden of many dangerous infectious diseases, such as smallpox, poliomyelitis, diphtheria, tetanus and measles [3]. Faced with the epidemic of COVID-19, researchers have been racing to develop and test effective vaccines against COVID-19 that mimic the host immune response to the pathogen and elicit the activation of T cells and antibody production, leading to high efficacy in constraining this infectious disease. To date, several highly effective COVID-19 vaccines have been approved for use in humans or are still in clinical development worldwide [4]. The developed COVID-19 vaccines were shown to have > 90% efficacy and thus could protect most vaccinated individuals [5]. However, a considerable proportion of individuals still suffer from SARS-CoV-2 infection and the associated COVID-19 for several reasons, including limited global vaccine acceptance, inequitable global distribution of vaccines, limited cross-protection, the short duration of protection, virus variants, and individual immunosuppression [6]. Similarly, other infectious diseases continue to be main cause of mortality including HPV, HBV, HIV, HCV, influenza virus and so on (Fig. 1) [7]. Chronic non-communicable diseases represent a major source of morbidity and mortality in worldwide, resulting 71% of all deaths and serious global economic burden (Fig. 1) [8]. Chronic non-communicable diseases mainly include cardiovascular disease, cancer, respiratory diseases, diabetes, hypertension, Alzheimer’s disease, dyslipidemia, asthma, chronic obstructive and pulmonary disease [9]. The top four chronic non-communicable disease killers account for more than 80% of all deaths including cardiovascular diseases, cancers, respiratory diseases, and diabetes [8].

Therefore, there is a need for therapeutic vaccines that can break the body’s immune tolerance and enhance the body’s specific immune response for the purpose of eradicating an established disease. Therapeutic vaccines are used to stimulate antigen-specific immune responses to specifically target and kill infected cells [10]. In 1890, Koch developed a therapeutic vaccine for tuberculosis containing tuberculin and glycerol in suspension, setting a precedent for therapeutic vaccines, and proposed that vaccines can not only prevent diseases but also treat diseases [11]. After Almroth Wright reported the use of a corresponding antibacterial vaccine to treat long-term local bacterial infections in 1897, therapeutic vaccines entered a period of great development but also great controversy due to abuse [12]. Due to the advent of various chemicals and antibiotics, researchers in the development of therapeutic vaccines have been frustrated. Since the discovery of HIV in 1981, the basis for and application of antiviral immunology have developed rapidly. The increasing number of patients with chronic diseases and the emergence of antibiotic resistance have led to the redevelopment of therapeutic vaccines. Following the approval of Sipuleucel-T in 2010 [13], therapeutic vaccine development entered a booming stage. A number of studies have shown that therapeutic vaccines play a positive clinical role in the treatment of tumors and infectious diseases [14, 15], and an increasing number of therapeutic vaccines have been transferred from basic laboratory research to clinical trials. In fact, there are several therapeutic vaccines in clinical trials against infectious disease and chronic non-communicable diseases such as virus infection, cancer [16], hypertension, Alzheimer’s disease, diabetes and dyslipidemia [14]. Compared with current chemical drugs or other biological drugs, therapeutic vaccines have the advantages of high specificity, few side effects, long-lasting effects, and no drug resistance, making them a new hope for the treatment of infectious diseases and chronic noncommunicable diseases [17]. In this review, we have provided the development of therapeutic vaccines on some infectious diseases and chronic noncommunicable diseases.

## Therapeutic vaccine-induced immunity

Researchers are racing to develop and test such effective prophylactic vaccines against infectious diseases that mimic the immune response against pathogens and elicit the activation of T cells and antibody production, leading to a highly efficacious constraint of infectious diseases. Prophylactic vaccines against hepatitis B virus (HBV) and human papillomavirus (HPV) have achieved great success [18, 19]. However, some viruses are able to cause persistent infection in some humans, for example, individuals at high risk for HPV infection. HPV has developed various approaches to escape immune surveillance, such as the low expression of viral proteins and the inhibition of antiviral immunity by suppressing APC function and the expression of MHC I molecules [20]. Similarly, cancer cells fail to be cleared by the immune response due to the low immunogenicity of the tumor antigen, the elimination of high-affinity T cells recognizing self-antigens and the immunosuppressive tumor microenvironment [21]. The ability of therapeutic vaccines to activate and amplify antigen-specific immune responses has been recognized as a potentially powerful tool for established diseases. The concept of therapeutic vaccines is based on the constant or unique expression of specific antigens, such as HPV viral E6 and E7, tumor neoantigens, and HBsAg, in established diseases [22–24]. Early, Saveria Campo et al. observed that an E7 protein vaccine against bovine papillomavirus type 4 induced a strong cellular immune response and resulted in the rejection of established tumors [25].

**Fig. 2:**
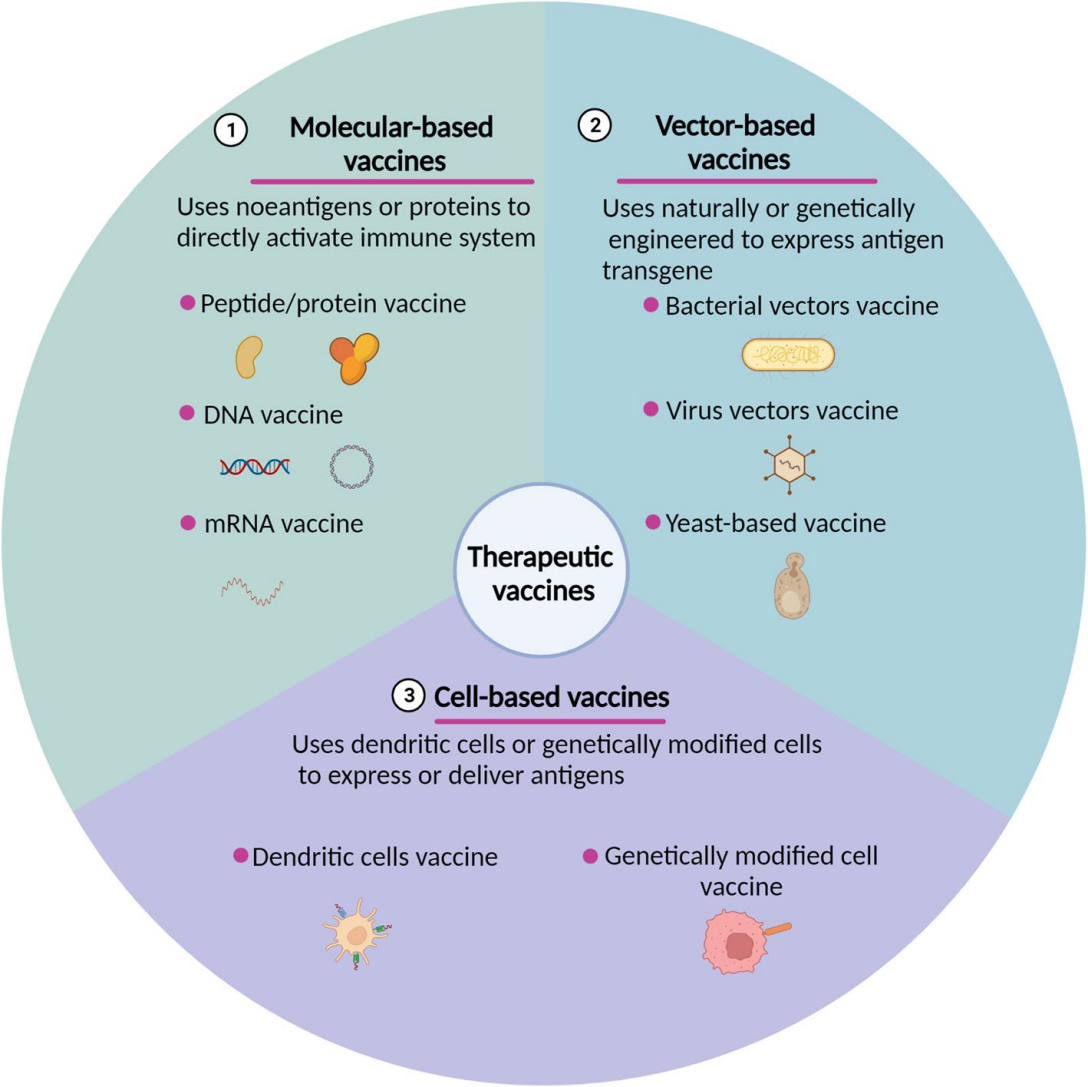
An overview of therapeutic vaccine types in preclinical and clinical trials. Therapeutic vaccines have several types used in preclinical and clinical phase including molecular-based vaccines (peptide/protein vaccine, DNA and mRNA vaccine), vector-based vaccines (bacterial vectors vaccine, viral vectors vaccine and yeast-based vaccines) and cell-based vaccines (dendritic cells vaccines and genetically modified cell vaccines)

**Fig. 3:**
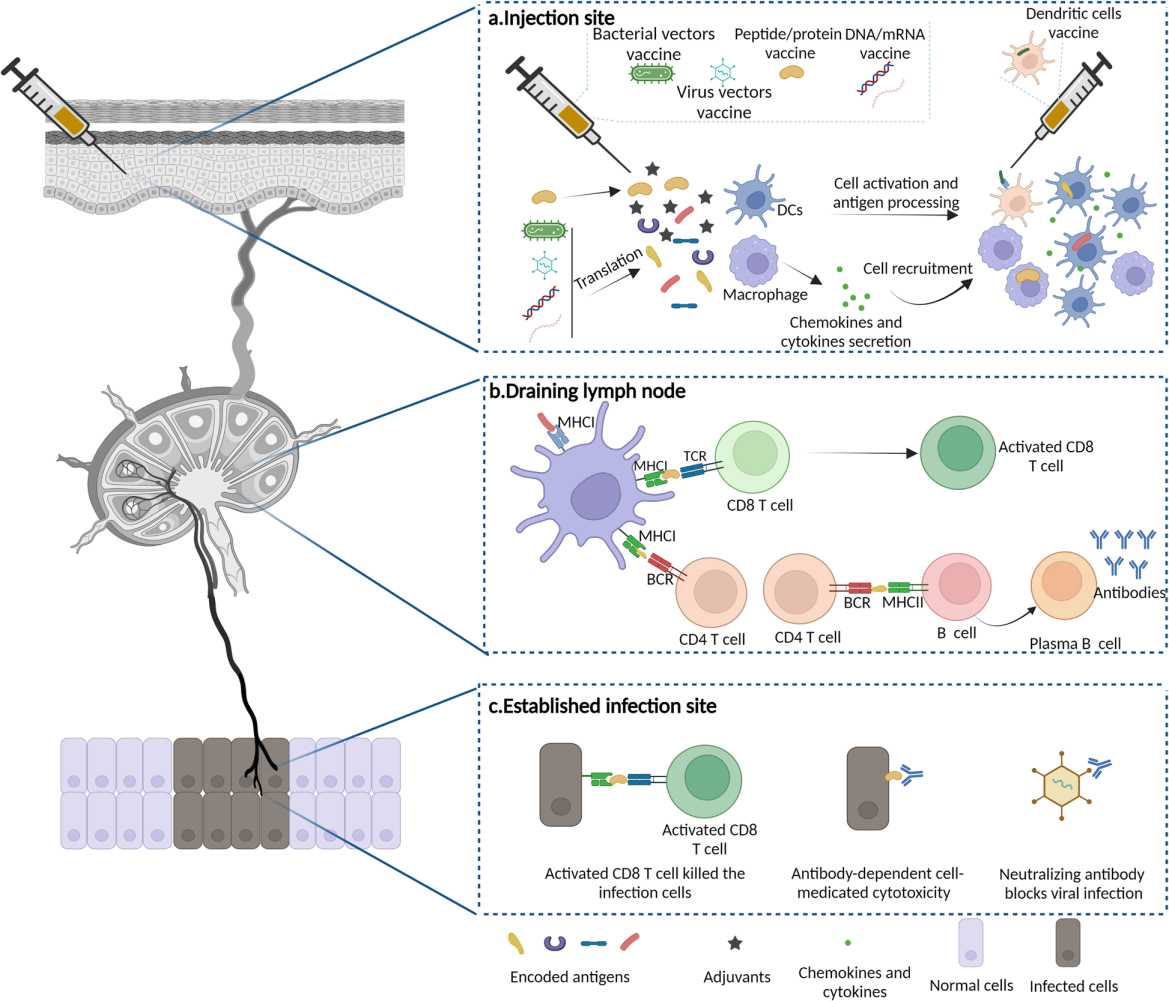
Therapeutic vaccine immunity. **a** Therapeutic vaccine, such as including bacterial vectors vaccine, viral vectors vaccine, peptide/protein vaccine, DNA and mRNA vaccine were injected locally. Adjuvants in the vaccine in local sites activate the resident innate immune cells and result in the release of chemokines (CCL2，CXCL1 et al) and cytokines, which subsequently recruits macrophages and DCs to the site of injection. The encoded antigen of the vaccine in the injection site is endocytosed by DCs; **b** Activated DCs in the vaccination sites travels to the draining lymph nodes. Upregulation of MHCII and co-stimulatory molecules such as CD40, CD80, and CD86 on the surface of DCs and cytokines are essential for DCs activation. Migratory and activated DCs present antigen in form of peptide-MHC complexes directly to T cells in the lymph nodes. Meanwhile receiving the co-stimulatory and cytokine signals, CD8^+^ T cells are activated. Antigen-activated B undergo maturation and expressed antigen-specific antibodies; **c** Antigen-specific CD8^+^ T cells and antigen-specific antibodies infiltrated into infection and lesions to conduct the function

Therapeutic vaccines are mainly divided into three types used in preclinical and clinical phases: molecular-based vaccines, vector-based vaccines and cell-based vaccines (Fig. 2) [26]. Molecular-based vaccines include peptide/protein vaccines, DNA vaccines and mRNA vaccines, applying neoantigens, purified peptides/proteins or DNA/mRNA-encoded proteins with adjuvants to trigger immune responses [27]. Vector-based vaccines use naturally or genetically engineered bacteria, viruses, and yeast as effective carriers to express antigen transgenes [28, 29]. Cell-based vaccines consist of dendritic cell vaccines and genetically modified cell vaccines, which use dendritic cells or genetically modified cells to express or deliver antigens [30, 31]. Therapeutic vaccines typically involve endogenous or exogenous antigen delivery, in most cases, with an adjuvant to activate dendritic cells (DCs). The purpose of therapeutic vaccines is to target the existing antigens to maximize the induction of epitope-specific T cells that can reach the infection site and lesions to eliminate infection or B cells to produce specific antibodies that can neutralize the virus [32]. The vaccine immune response is complicated and occurs in multiple locations (Fig. 3).

Adjuvants are crucial components in vaccines to stimulate and enhance the magnitude and durability of the immune response against antigens [33]. Adjuvants in licensed vaccines include alum, MF59, CpG 1018, AS01, AS02, AS03 and AS04, and several high-potency adjuvants are being evaluated in preclinical and clinical studies. Adjuvants with immunostimulatory effects mimic damage-associated molecular patterns (DAMPs) or pathogen-associated molecular patterns (PAMPs) to activate innate immune cells through the stimulation of pattern recognition receptors (PRRs) [33]. Therapeutic vaccines are commonly injected via intramuscular, subcutaneous or intradermal routes in the clinic, with intramuscular administration being most frequently used [34]. Local inflammation plays a critical role in the magnitude and duration of the adaptive immune response [35]. At the vaccine delivery site, adjuvants in vaccines activate resident innate immune cells, resulting in the release of chemokines (*CCL2, CXCL1,* etc.) and cytokines, which subsequently recruit innate immune cells, including neutrophils, monocytes, macrophages and DCs, to the site of injection [36, 37]. DCs infiltrate the injection site and then serve as the main antigen-presenting cells (APCs), playing a vital role in eliciting a strong adaptive immune response [34]. The antigen component of the vaccine is endocytosed by DCs in the vaccination site.

DCs activated at the vaccination site and vaccine components travel to the draining lymph nodes [38]. The upregulation of MHCII and costimulatory molecules such as CD40, CD80, and CD86 on the surface of DCs and cytokines are essential for DC activation. Migratory and activated DCs present antigen in the form of peptide–MHC complexes directly to T cells in the lymph nodes. By receiving costimulatory and cytokine signals, CD8^+^ T cells are activated [39]. Antigen-activated B cells undergo maturation and rapid proliferation. Then, antigen-specific CD8^+^ T cells travel to infection sites and lesions to perform their function.

## Therapeutic vaccines against infectious diseases

Multiple of severe infectious diseases, not least the COVID-19 pandemic, had a devastating impact on individuals [40]. For example, HPV-related malignancies account for 4.5% of all human cancers [41]. It is estimated that approximately 257 million people have chronic HBV infection and thus an increased risk of developing liver cirrhosis and hepatocellular carcinoma [42]. There were approximately 38 million HIV infections who need life-long antiretroviral treatment [43]. Therapeutic strategies need to be developed to solve persistent virus infection and associated lesions in clinic. Here, we review the development of therapeutic vaccines for HPV, HBV, HIV, HCV and SARS-CoV-2. Selected clinical trials of therapeutic vaccines against infectious disease were should in Table 1.

**Table 1:** Selected clinical trials of therapeutic vaccines against infectious disease

Therapeutic HPV vaccines					
Vaccine type	Vaccine Formulation	Combination agents	Condition	Clinical trial identifier	Phase/status
Bacterial vector vaccine	ADXS11–001(attenuated live Listeria Encoding HPV 16 E7 vector)		Cervical carcinoma	NCT02164461	Phase I-II Completed
			Cervical carcinoma Head and Neck Cance	NCT02291055	Phase I-II Active, not recruiting
			Cervical cancer	NCT01266460	Phase II Completed
		Placebo	Cervical cancer	NCT02853604	Phase III Active, not recruiting
			Anal cancer Rectal cancer	NCT02399813	Phase II Completed
			Head and neck cancer, oropharyngeal squamous cell carcinoma	NCT02002182	Phase II Active, not recruiting
Viral vector vaccine	Vvax001(Semliki Forest virus expressing E6 and E7)		CIN 2/3 Cervical cancer	NCT03141463	Phase I Completed
	HB-201 (lymphocytic choriomeningitis virus encoding HPV E6 and E7)		HPV-Related Squamous Cell Carcinoma	NCT04180215	Phase I/II Recruiting
Peptide/protein vaccine	ISA101(nine HPV-16 E6 and four HPV-16 E7 synthetic peptides with Montanide ISA51)	Nivolumab	Solid Tumors	NCT02426892	Phase II Completed
	TVGV-1(HPV16 E7 fused protein +adjuvant GPI-0100)		High-grade squamous intraepithelial lesions	NCT02576561	Phase IIa Active, not recruiting
DNA vaccine	VGX-3100(plasmid encoding E6 and E7 of HPV16/18)		Head and neck squamous cell cancer	NCT02163057	Phase I/IIa Completed
			Cervical cancer	NCT02172911	Phase I/IIa Completed
		Placebo	High-grade squamous intraepithelial lesion of the cervix	NCT03185013	Phase III Completed
	GX188E (HPV E6/E7 fused to Flt3L		Cervical intraepithelial neoplasia	NCT02139267	Phase II Completed
		Placebo	Cervical Intraepithelial Neoplasia	NCT02596243	Phase II Active, not recruiting
			Cervical Intraepithelial Neoplasia 3	NCT03206138	Phase Ib/II Recruiting
Therapeutic HBV vaccines					
Viral vector vaccines	TG1050(Adeno vector encoding core, polymerase, envelope fusion protein)	Placebo	Chronic HBV Infection	NCT02428400	Phase I/Ib Completed
Protein vaccine	Theravax (DV-601, consisting of HBsAg, HBcAg and saponin-based ISCOMATRIX adjuvant)	Entecavir	Chronic HBV Infection	NCT01023230	Phase IIb Completed
	GS-4774(Heat-inactivated yeast containing S, core, X proteins)		Chronic HBV Infection	NCT01943799	Phase II Completed
	GS-4774(Heat-inactivated yeast containing S, core, X proteins)	Tenofovir	Chronic HBV Infection	NCT02174276	Phase II Completed
	HeberNasvac (containing HBsAg and HBcAg)	Peg-IFN	Chronic HBV Infection	NCT01374308	Phase II Completed
	HeberNasvac (containing HBsAg and HBcAg)	NUC	Chronic HBV Infection	NCT02249988	Phase IIB-III Completed
	HepTcell vaccine (Synthetic peptide+ IC31 adjuvant)		Chronic HBV Infection	NCT02496897	Phase I Completed
DNA vaccine	INO-1800 (DNA plasmids encoding S and core)	NUC	Chronic HBV Infection	NCT02431312	Phase I Completed
	HB-110(a mixed plasmid DNA)	Adefovir	Chronic HBV Infection	NCT00513968	Phase I Completed
		Adefovir	Chronic HBV Infection	NCT01641536	Phase I Completed
Therapeutic HIV vaccines					
DC-based vaccines	AGS-004(encoding the autologous HIV antigens Gag, Nef, Rev., and Vpr)	–	HIV Infection	NCT00672191	Phase II Completed
	HIV-1 ApB	–	HIV Infection	NCT00510497	Phase I/II Completed
mRNA vaccines	iHIVARNA (consisting of HIV immunogen sequence and a mixture of activation molecules (CD40L, CD70 and caTLR4))	–	HIV-infection	NCT02413645	Phase I Completed
		–	HIV-infection	NCT02888756	Phase IIa Terminated
Viral vector vaccines	MVA.HIVconsv(encoding 14 highly conserved regions of the viral proteome)	–	HIV-I infection	NCT01024842	Phase I Terminated
	HIVAX	–	HIV-infection	NCT01428596	Phase I
Therapeutic HCV vaccines					
Yeast vector vaccine	GI-5005(expressing an NS3-core fusion protein)	–	HCV infection	NCT00124215	Phase I Completed
		–	HCV infection	NCT00606086	Phase II Completed
Viral vector vaccines	AdCh3NSmut/ Ad6NSmut (encoding HCV proteins)	–	HCV infection	NCT01094873	Phase I
	TG4040(expressing NS3/4/5B proteins)	–	HCV infection	NCT01055821	Phase I Completed

Source: The clinical trials were from ClinicalTrials.gov

### Therapeutic vaccines against HPV

Human papillomavirus (HPV) is recognized as the main cause of cervical cancer, the 4th most common cancer in women, accounting for 99.7% of cervical cancer cases and a subset of other diseases, such as vulvar, vaginal, penile, and anal cancers and head and neck cancers [44]. Persistent infection with HPVs precedes the development of high-grade squamous intraepithelial lesions, which can progress to malignant cancers [45]. It is estimated that HPVs cause 275,000 deaths from cervical cancer and result in 530,000 new cases every year, becoming a serious public health problem worldwide and causing a loss of life [46]. To decrease the prevalence of HPV-associated diseases, the development of prophylactic vaccines has been emphasized, and these vaccines have shown promising efficacy. Three commercially available HPV prophylactic vaccines, Cervarix, Gardasil and Gardasil 9, target several types of HPVs by producing neutralizing antibodies and preventing HPV infections and precancerous cervical lesions with almost 100% efficacy [47]. However, the incidence of HPV-related tumors remains high for several different reasons, including the low vaccination rate, lack of targeting all types of HPV, age-based recommended immunization practices and high costs of immunization [48]. More importantly, infected individuals cannot benefit from prophylactic vaccines [49]. Therefore, therapeutic vaccines against more types of oncogenic HPV are urgently needed for the HPV-infected population.

Therapeutic HPV vaccines mainly aim to expand or induce strong specific Th1-type and CTL responses to kill infected cells. An ideal antigen for a therapeutic vaccine against established HPV infections and HPV-associated lesions should have the qualities of being essential for the onset and maintenance of malignancy, constitutive expression at high levels and an absence of mutation. The HPV E6 and E7 oncoproteins represent near-ideal targets for the development of most HPV therapeutic vaccines, such as bacteria-, virus-, peptide-, DNA- and DC-based vaccines [50].

Bacterial vectors, including *Listeria monocytogenes*, *Lactobacillus casei*, *Lactobacillus lactis* and *Salmonella* vectors, have natural adjuvant properties and have the ability to modulate antigen presentation through MHC I and MHC II pathways, activating CD8^+^ T cells and CD4^+^ T cells [10]. ADXS11–001 is a live attenuated *L. monocytogenes (Lm)* which was modified to express HPV16 E7 joined to the protein listeriolysin-O (LLO) [51]. LLO contributes to the replication of *Lm* in APCs which allows antigens secreted by *Lm* and presented by APCs [52]. ADXS11–001 showed an acceptable safety profile and efficacy in activating HPV16 E7-specific T-cell responses in patients with invasive carcinoma of the cervix in a phase I clinical trial [51]. A phase II study showed increased survival in patients with advanced cervical cancer, with a 12-month combined survival rate of 34.9%, exceeding the historical overall survival [53]. Based on the encouraging data, a phase III trial is being conducted for advanced cervical cancer (NCT02653604). GLBL101c composited with an *L. casei* bacterial vector secreting full-length HPV16 E7 protein elicited E7-specific mucosal immunity and resulted in pathological downgrades in the cervix of CIN3 patients [54]. Another therapeutic vaccine based on *L. casei*, named NZ8123-HPV16-optiE6, was safe, increased the production of antibody and activated E6-specific IFN-γ-secreting CD8^+^ CTL responses in a phase I trial [55].

Virus vectors, including adenovirus, adeno-associated virus, alphavirus, and modified vaccinia Ankara (MVA) viral vectors, can be used to deliver antigens to induce an immune response [56]. In clinical trials, an MVA vector was used to express HPV16/18 E6 and E7 proteins, forming a TA-HPV vaccine. The TA-HPV vaccine was safe and immunogenic and generated HPV-specific CTL responses in phase I/II and II trials [57, 58]. More recently, Tipapkinogen Sovacivec (TS), an MVA-based vaccine that encodes human cytokine IL-2, HPV16 E6 and E7 proteins, significantly cleared viral DNA and achieved greater complete resolution rates of histological CIN3 disease [59]. Human adenovirus, another commonly used viral vector, was used to express HPV E6 and E7 to prepare the vaccine [60]. To avoid preexisting immunity against human adenovirus, a chimpanzee adenovirus vector was alternatively designed to deliver HPV antigens, and the efficacy alone or in combination with anti-PDL1/TGF-beta Trap is being tested in a clinical trial (NCT04432597). In addition to DNA viruses, RNA viral vectors have also been designed as antigen transporters. Vvax001, a Semliki Forest virus expressing E6 and E7, efficiently induces long-term CTL activity and a potent therapeutic antitumor effect in mice [61]. The phase I trial of Vvax001 was completed, but the results were not reported (NCT03141463). HB-201, encoding HPV E6 and E7, which is based on the arenavirus lymphocytic choriomeningitis virus, showed excellent therapeutic efficacy in a preclinical model [62]. Currently, a phase I/II study of Vvax001 in HPV16^+^ recurrent/metastatic head and neck squamous cell carcinoma is launching (NCT04180215).

Peptide-based vaccines are MHC-specific and easy to manufacture. However, adjuvants such as cytokines and TLR ligands are often mixed with peptide-based vaccines to enhance CD8^+^ T-cell responses due to the low antigenicity of peptides [63]. An early peptide vaccine consisting of HPV16 E7 _12–20/86–93_ epitopes and incomplete Freund’s adjuvant demonstrated decent biological and clinical effects [64]. ISA101 is an HPV16 vaccine consisting of nine HPV16 E6 and four HPV16 E7 synthetic peptides with the adjuvant Montanide ISA51 [65]. In the phase II study in women with high-grade vulvar intraepithelial neoplasia, clinical responses were observed in 15 of 19 patients (79%), and a complete response was observed in 9 of 19 patients (47%). Moreover, all patients had the capacity to develop vaccine-induced T-cell responses. The stronger CD4^+^ and CD8^+^ T-cell IFN-γ responses in patients contributed to the complete response [65]. Multiple studies have also demonstrated the excellent clinical effect of ISA101 [66–70]. To further augment the efficacy of ISA101, the combination of ISA101 and nivolumab showed promise in long-term follow-up (NCT02426892) [71]. The phase II trial of nivolumab and ISA101 vaccination showed a median overall survival of 15.2 months and 2-year overall survival rate of 33% among patients with incurable HPV16 ^+^ cancer. Moreover, the infiltration of cytotoxic T cells in tumors and the activation of the interferon signaling pathway strongly benefited the clinical response [71]. Additionally, a randomized phase II trial of ISA101 and cemiplimab (PD-1 blocking antibody) is ongoing (NCT03669718).

Compared to peptide-based vaccines, protein-based vaccines contain more epitopes and can induce memory CD8^+^ T-cell responses but show a preference for eliciting humoral immunity. Protein antigens are often designed to fuse with other proteins to expand antigen-specific immunity. GTL001 is formed by fusing the E7 proteins of HPV16 and HPV18 E7 proteins to catalytically inactive *Bordetella pertussis* CyaA, which specifically delivers E7 to CD11b^+^ antigen-presenting cells [72]. In a phase I trial (EudraCT No. 2010–018629-21), GTL001 in conjunction with imiquimod significantly reduced the viral load of HPV16/18 in patients infected by HPV16 or HPV18 [72]. A similar strategy was used to design SCN-00101, which fused HPV16 E7 protein to *M. bovis* BCG heat shock protein [73]. Of the 58 patients treated with SCN-00101 in the phase II trial (NCT00075569), 13 (22.5%) had a complete pathological response, 32 (55%) had a partial response and 11 (19%) had stable disease [73]. Recently, Da Silva et al. reported another fused protein vaccine, TVGV-1, which consisted of HPV16 E7 protein covalently linked to a bacterial exotoxin and an endoplasmic reticulum retention signal [74]. TVGV-1 significantly activated E7-specific CD8^+^ T-cell immunity in a mouse tumor model [74]. Based on the promising effect in vitro, the safety and efficacy of the TVGV-1 vaccine were assessed in a phase IIa clinical trial (NCT02576561).

DNA vaccinations consist of direct injection of plasmid DNA encoding antigens into host tissue, achieving sustained antigen expression. DNA vaccines do not induce neutralizing antibodies against the vector, allowing repeated vaccination [75]. Moreover, DNA vaccines are easily manufactured and can stimulate humoral and cell-mediated immunological responses, therefore serving as desirable therapeutic vaccine strategies. However, the poor immunogenicity and the risk of DNA integration into chromosomes remain the main drawbacks of DNA vaccines [76]. The immunogenicity of VGX-3100, a synthetic DNA vaccine in which the plasmid encodes the E6 and E7 proteins of HPV16/18, was assessed in women with CIN2/3 (NCT00685412) in a phase I clinical trial [77]. Overall, 78% of patients generated increased Th1-biased cellular immune responses. Based on the promising results, the efficacy, safety, and immunogenicity of VGX-3100 were further assessed in women with HPV16/18 and CIN2/3 (NCT01304524) in a phase II clinical trial [78]. In the per-protocol analysis, a total of 53/107 (49.5%) patients treated with VGX-3100 experienced histopathological regression, compared to 11/36 (30.6%) patients treated with placebo. Then, in the intention-to-treat analysis, increased histopathological regression among the VGX-3100 group (48.2%) compared with the placebo group (30.0%) was observed. A randomized, double-blind, placebo-controlled phase III study to determine the efficacy, safety, and tolerability of VGX-3100 in adult women with CIN2/3 associated with HPV16/18 was completed in 2021 (NCT03185013). However, the results have not been reported. Similarly, DNA vaccines have been rationally designed to link antigens with other proteins to enhance antigen immunogenicity. GX188E was engineered to coexpress HPV E6/E7 by fusing to Fms-like tyrosine kinase-3 ligand (Flt3L), which aimed to promote antigen presentation and trafficking [79]. In a clinical phase I trial (NCT01634503), nine patients with CIN3 received GX-188E by electroporation. A significant E6/E7-specific Th1-type cellular immune response was observed in all nine patients. Moreover, 7/9 (78%) patients showed a polyfunctional HPV16-specific CD8 T-cell response, which contributed to HPV clearance and a histological CR [79]. The efficacy of GX-188E for inducing the regression of CIN3 was further determined in a randomized, open-label, phase II trial (NCT02139267) [80]. After receiving the GX-188E vaccine, histopathological regression was observed in 52% (33/64) of patients in the per-protocol analysis and 67% (35/52) of patients in the extension analysis. Importantly, 73% of the patients with histological regression in the per-protocol analysis and 77% in the extension analysis showed HPV clearance, which was associated with enhanced IFN-γ production [80]. Other DNA vaccines, such as pNGVL4a-CRT-E7 [81] and ZYC101 [82], have also exhibited good efficacy and safety in phase I clinical trials.

### Therapeutic vaccines for HBV

Chronic hepatitis B virus (HBV) infection has been considered a worldwide public health issue. HBV is a partially double-stranded DNA virus belonging to the Hepadnaviridae family that exclusively infects hepatocytes [83]. HBV prophylactic vaccination is safe and has been accepted worldwide as an effective method to avoid HBV infection. It was demonstrated that approximately 90% of followed individuals who received a usual three-dose HBV prophylactic vaccine remained protected for ≥30 years [84]. However, some people are still infected by HBV for several reasons, such as unresponsiveness to HBV vaccination, HBV mutants, and no opportunity for vaccination [85]. Once a chronic HBV infection is established, most people will remain infected for life, which largely increases the risk of liver-related death.

For the majority of affected individuals, the first-line therapies for the treatment of chronic HBV (CHB) infection are third-generation nucleot(s) ide analogs (NUCs) and interferon-alpha (IFN), which effectively improve the quality and duration of life by preventing progression to underlying liver disease [86]. Short-term treatment with IFN leads to a sustained virological response and subsequent HBsAg loss in only approximately 30% of patients and is frequently associated with poor tolerability, side effects and low effectiveness [87, 88]. Conversely, NUC therapy leads to the suppression of HBV replication in almost all treated patients; however, long-term administration is required to avoid virus reactivation after stopping treatment [89]. Therefore, there is an urgent need to develop new drugs to shorten the therapy administration time and achieve an HBV cure.

The main reason for HBV persistence after treatment with multiple drugs is dysfunctional HBV-specific T and B cells, with characteristics of low frequency, functional defects and exhaustion [90–92]. Therapeutic vaccination aims to reconstitute the systemic immune system and elicit or augment existing HBV-specific B and T-cell responses, representing a rational strategy to overcome immune tolerance and cure chronically HBV-infected patients. Therapeutic vaccination represents an attractive option for CHB infection therapy. At present, several different therapeutic vaccines have been assessed in clinical trials, including protein- or peptide-based, DNA- and viral vector-based vaccines.

Adenovirus induces several strong innate immune signaling pathways and subsequently effectively activates robust adaptive humoral and cellular immune responses; therefore, it is currently being applied in cancer vaccines [93]. TG1050, consisting of a nonreplicative adenoviral vector encoding HBV core, polymerase and envelope domains, is a promising vaccine candidate [94]. TG1050 effectively activated the cytolytic activity of T cells and exerted an antiviral effect in HBV-naïve and HBV-persistent mouse models [94].

The safety, immunogenicity and early efficacy of TG1050 in CHB patients were assessed in a phase I clinical trial (NCT02428400) [95]. IFN-γ-producing T cells and minor decreases in HBsAg were observed after TG1050 vaccination. Interestingly, the HBV-specific cellular immune response was enhanced by the combination of an NUC and TG1050 [95]. Additionally, some new strategies have been utilized to clear HBV with adenovirus vectors. For example, adenovirus was designed to deliver a CRISPR/Cas9 system and a single guide RNA (gRNA) system to degrade the HBV genome [96, 97].

Another effective virus vector was designed using a modified vaccinia Ankara (MVA) viral vector for the delivery of antigens [98]. To improve therapeutic vaccine efficacy, a novel HBV vaccine that consisted of chimpanzee adenovirus and MVA viral vectors encoding multiple HBV antigens administered through a prime-boost strategy was proposed [99]. Adenovirus prime followed by MVA boost vaccination coordinately enhanced polyfunctional HBV-specific CD8^+^ and CD4^+^ T-cell responses in mice [99].

Among the candidate vaccines, protein-based vaccines have attracted some attention. Theravax (DV-601) comprises recombinant HBV surface antigen (HBsAg) and HBV core antigen (HcAg), with a saponin-based ISCOMATRIX adjuvant. Theravax vaccination led to the development of an HBV-specific lymphoproliferative response, an HBc-specific interferon-gamma T-cell response and a reduction in HBV DNA [100]. Another protein-based vaccine, GS-4774, is a heat-inactivated and engineered yeast-based vaccine that recombinantly expresses a fusion protein consisting of HBsAg, HBcAg and HBx [101]. The yeast component exerts a strong adjuvant effect by enhancing DC presentation and eliciting a significant T-cell response [102]. GS-4774 was safe and well tolerated in healthy participants (NCT01779505). Then, the efficacy of GS-4774 as a therapeutic vaccine was tested in the phase II trial NCT01943799 in 178 virally suppressed patients with chronic hepatitis B infection. In the study, GS-4774 did not provide significant reductions in serum HBsAg, and only three patients had HBsAg declines ≥0.5 log_10_ IU/ml after receiving the highest vaccine dose. Although low HBV-specific T-cell responses were detected in all patients, GS-4774 did not result in a clinical benefit. To obtain a therapeutic benefit, GS-4774 was combined with tenofovir therapy in patients with chronic hepatitis (NCT02174276). Although HBsAg reduction or loss was not observed in participants, GS-4774 induced a strong CD8^+^ T-cell immune stimulatory effect, which paved the way for combination with other therapies, such as silencing RNA compounds and ICIs and modulating T-cell metabolism [103].

Among the new peptide-based vaccine candidates evaluated in clinical trials, HeberNasvac contained both HBsAg and HBcAg [104]. HeberNasvac showed safety, good tolerance and immunogenicity in a phase I study in healthy adults [105]. The safety and the ability to control the virus were further confirmed in subsequent clinical trials [105]. In a phase III trial of HeberNasvac versus Peg-IFN, HeberNasvac induced a superior reduction in HBV DNA load under the limit of detection (NCT01374308) [106]. Based on the clinical results, HeberNasvac was approved by the Cuban Regulatory Authority at the end of 2015. The phase IIB-III efficacy study was assessed as an adjunct therapy to NUCs to control HBV replication (NCT02249988). The HepTcell vaccine is formed by nine synthetic CD4^+^ and CD8^+^ T-cell peptides derived from the most conserved domains of HBV with an IC31 adjuvant (NCT02496897) [24].

DNA-based vaccines have also been studied and evaluated in clinical trials for CHB therapeutic vaccination. INO-1800 is a synthetic DNA vaccine that encodes HBsAg and the consensus sequence of HBcAg, and it induced a strong antigen-specific T-cell and B-cell response in immunized mice [107]. The addition of INO-9112, a plasmid expressing IL-12, to INO-1800 activated cytotoxic T lymphocytes, showing the efficacy of these synthetic plasmids as components of therapeutic HBV vaccines [108]. Another candidate vaccine, HB-110, is composed of plasmids encoding HBs, PreS1, HBc, Hbpol and IL-12 [109]. Higher T-cell and antibody responses were observed in mice [110].

### Therapeutic vaccines for human immunodeficiency virus (HIV)

The first report of AIDS caused by HIV was published in 1981, and globally, there were approximately 38 million HIV infections and a total of 690,000 AIDS-related deaths in 2020 [43]. Most infected individuals need life-long antiretroviral treatment, which highlights the urgent need for a prophylactic HIV vaccine to prevent infection. Over the past 40 years, great efforts have been made in the development of HIV vaccines, but no effective vaccine has been approved. The main hurdles in developing an effective HIV vaccine include the high variability of HIV, genetic diversity and a lack of understanding about immune protection [111]. Among the many HIV vaccine candidates, RV-144 was the only vaccine that was tested in a clinical trial and achieved 31.2% efficacy in Thailand [112]. The failure of HIV prophylactic vaccine studies motivated the design idea of creating therapeutic vaccines to combat HIV. DNA, peptide, protein, viral, mRNA and DC vaccines have been tested in clinical trials. Protein-, peptide-, and DNA-based vaccines aim to induce cellular immunity and humoral immunity to viral proteins, but these vaccines have yet to provide effective treatment. Consequently, we mainly discuss DC-, mRNA- and viral-based vaccines.

DC-based vaccines are promising vaccine candidates that play a vital role in inducing an immune response against antigens [113]. Generally, autologous DCs are isolated from patients, loaded with antigens in vitro and returned to patients [114].AGS-004 is an autologous DC vaccine that was loaded in vitro with RNA encoding the autologous HIV antigens Gag, Nef, Rev., and Vpr [115]. The activity of AGS-004 was tested in a phase IIB study in which 54 HIV-1-infected patients were enrolled (NCT00672191) [116]. AGS-004 elicited an HIV-specific effector/memory CD8 T-cell response but showed no antiviral effect. Another study enrolled six male individuals to evaluate the immunogenicity of AGS-004 [115]. Multifunctional HIV-1-specific effector/memory CTLs were induced in all participants, which was strongly related to a longer time to viral rebound. The HIV-1 ApB DC vaccine also contained autologous DCs loaded with autologous HIV-1-infected apoptotic cells. The vaccine was safe and well tolerated in a phase I/II clinical trial but did not prevent viral rebound during treatment interruption (NCT00510497) [114].

The administration of naked mRNA represents a promising alternative to immunogens. Leal et al. performed a first-in-human phase I clinical trial with mRNA-based vaccines (NCT02413645) [117]. The naked mRNA in the vaccine consisted of a novel HIV immunogen sequence and a mixture of activation molecules (CD40L, CD70 and caTLR4). The safety and efficacy of the three intranodal doses of mRNA vaccine were evaluated in 21 patients with chronic HIV-1 infection. The results demonstrated that the mRNA vaccine was safe and activated HIV-specific T-cell responses. However, interim analysis did not show sufficient immunogenicity of IMP compared to placebo in a phase IIa study of the mRNA vaccine (NCT02888756) [118].

Recombinant viral vectors readily achieved intracellular antigen expression to induce a CTL response [119]. The MVA.HIVconsv vaccine consisted of a modified vaccinia Ankara (MVA) viral vector, which encodes a chimeric protein comprising 14 highly conserved regions of the viral proteome [120]. The immunogenicity and activity of the MVA.HIVconsv vaccine was tested in clinical trials (NCT01024842) [120]. Although the MVA.HIVconsv vaccine was safe in HIV-positive patients, and it displayed modest immunogenicity and weak antiviral activity. HIVAX is a replication-defective HIV-1 lentiviral vector vaccine that contains multiple mutations in its viral genome [121]. HIVAX enhanced the functionality of T cells and reduced the median viral load in HIV-1-infected participants (NCT01428596).

### Therapeutic vaccines for HCV

Hepatitis C virus (HCV) is a major causative factor of chronic liver disease worldwide, and approximately 2 million people are newly infected every year [122]. Upon infection, HCV can initiate a strong, broad, and persistent antigen-specific T-cell response, leading to a state of chronic hepatic inflammation, even liver cirrhosis and primary liver cancer (hepatocellular carcinoma, HCC) [123]. The approval of novel HCV-specific direct-acting antiviral (DAA) drugs improved the treatment of HCV but was associated with a number of side effects [124]. Therefore, it is important to develop therapeutic vaccines to treat HCV to lower the chronicity rate and the disease burden. Studies have found that CTLs and CD4^+^ T cells recognize the majority of the viral epitopes in patients with HCV infection via the NS3 region and NS5A/B [125].

Peptide/protein vaccines can be generated relatively easily and are being developed for infectious diseases. Peptide vaccines are HLA-specific and present vaccine peptides to T-cell receptors via HLA molecules. The efficacy of peptide vaccines targeting E1, E2, NS3 and NS5A was evaluated in a phase I trial. Fifty percent of participants produced peptide-specific IFN-γ by CTLs, but only 25% of HCV RNA was reduced [126]. IC41, a peptide vaccine composed of five synthetic peptides derived from the core, NS3, and NS4 proteins of HCV genotypes 1 and 2, with a poly-L-arginine adjuvant, induced significant immunological responses in 128 HLA-A2^+^ healthy volunteers in a phase I trial. However, the T-cell responses were too weak to induce a decrease in HCV RNA in the serum of most of the volunteers [127]. The suboptimal immune response was most likely due to the low immunogenicity of synthetic peptides. In a randomized trial, intensified dosing and intradermal (i.d.) administration of IC41 could induce more robust peptide-specific immune responses than in a previous study [128]. Another peptide vaccine, consisting of HCV core region (C35–44) peptides with emulsified incomplete Freund’s adjuvant (ISA51), was shown to be safe and well tolerated in a phase I trial [129]. Furthermore, Pevion Biotech developed a virosome-based vaccine containing NS3 peptides, and a phase I study of this vaccine was recently completed, but data have not been released (NCT00445419).

GI-5005, a yeast vector vaccine expressing an NS3-core fusion protein for HCV [130], induced effective NS3- and core-specific cellular immune responses in both C57BL/6 and BALB/c mice [131]. A phase I clinical trial showed that GI-5005 was well tolerated and was able to induce a significant HCV-specific immune response in patients (NCT00124215). A phase II trial aiming to investigate the treatment effect of combining GI-5005 and standard-of-care treatment has been completed (NCT00606086).

Plasmid DNA encoding antigenic HCV protein(s) or epitope(s) can induce both humoral and cellular immune responses in vivo [132]. DNA vaccines include the nucleotides encoding structural proteins or nonstructural proteins, such as NS3, NS4, NS5, core, and the envelope proteins E1/E2 [133]. CIGB-230, containing a mixture of core/E1/E2-expressing plasmids, was the first therapeutic DNA vaccine for HCV evaluated in clinical trials [134, 135]. The second HCV DNA-based vaccine, now in phase II clinical trials, developed for HCV infection was ChronVac-C, which includes the most conserved regions (NS3 and NS4a) [136]. Initial results suggested the safety and immunogenicity of the vaccine, and it is now in phase II clinical trials for HCV infection (NCT01335711). Ratnoglik et al. constructed a series of DNA vaccines that express NS3 with mutations in the catalytic triad of the serine protease and the NTPase/RNA helicase domain [137], which overcame the effects of NS3 on normal cell function [138].

The use of viral vectors for the delivery of HCV RNA is an appealing vaccine choice. Replication-defective adenovirus (Ads) and the nonreplicative modified vaccinia Ankara (MVA) virus are commonly used vectors for HCV viral-based vaccines. An Ad6-based vaccine encoding NS3, NS4A, NS4B, NS5A, and inactivated NS5B can induce specific T-cell responses against the NS antigens of HCV in mice, rhesus macaques, and even chimpanzees [139, 140]. The vaccine was found to be safe and immunologically potent in a phase I clinical trial (NCT01094873). An MVA-based therapeutic vaccine (TG4040) that expresses NS3/4/5B proteins can induce potent, long-lasting and in vivo cross-reactive T-cell responses [141]. TG4040 in combination with standard PEG-IFN and ribavirin therapy is being evaluated in a phase II clinical trial (NCT01055821).

Recently, the results of a clinical trial of DC treatment among patients with chronic HCV infection were reported. DCs loaded and activated ex vivo with HCV-specific HLA-A2 restricted T-cell epitope were injected intradermally into patients. All six patients who received the vaccine exhibited weak HCV-specific CD8^+^ T-cell responses. The results showed the safety but weak efficacy of the DC vaccine. To improve the efficacy of treatment, alternative dosing regimens or vaccination routes should be considered [142].

### Therapeutic effect of COVID-19 vaccines against SARS-CoV-2 infection

In addition to preventing the spread of SARS-CoV-2, multiple vaccines have been developed among countries worldwide, such as the CoronaVac, BNT162b2, Ad26.COV2.S, ChAdOx1 nCoV-19, mRNA-1273 and NVX-CoV2373 vaccines, against COVID-19 [143]. These vaccines activate the immune system and generate significantly high neutralizing antibodies against the virus, being highly efficacious in preventing SARS-CoV-2 infection [144]. Although millions of people have been infected and breakthrough infections (infections in fully vaccinated people [145]) do occur, COVID-19 vaccines are able to induce immunity to reduce persistent infection and severe disease, as well as hospitalizations and deaths [146].

Bradley et al. evaluated the efficacy of therapeutic mRNA vaccination in the context of persistent SARS-CoV-2 infection [147]. In this study, humoral and cellular responses were not detected in a 37-year-old Caucasian male with Wiskott-Aldrich syndrome after 120 days of PCR-confirmed SARS-CoV-2 infection. The immunodeficient man received two doses of the BNT162b2 mRNA COVID-19 vaccine one month apart. SARS-CoV-2-specific IFN-γ^+^ T cells and antibodies were increased at 14 days following the first vaccine dose. Interestingly, SARS-CoV-2 clearance was detected at 72 days following the first therapeutic vaccination. The researchers did not exclude viral clearance in an independent manner [147].

A study was conducted to evaluate the effectiveness of four COVID-19 vaccines (CoronaVac, ChAdOx1 nCoV-19, Ad26.COV2.S and BNT162b2) among individuals with previous SARS-CoV-2 infection in Brazil [148]. The vaccine effectiveness against symptomatic SARS-CoV-2 infection among the matched 22,566 individuals with previous infection was 39.4% for CoronaVac, 56.0% for ChAdOx1 nCoV-19, and 44.0% for Ad26.COV2.S, and 64.8% for BNT162b2. Furthermore, the effectiveness against hospitalization or death was 81.3% for CoronaVac, 89.9% for ChAdOx1 nCoV-19, 57.7% for Ad26.COV2.S, and 89.7% for BNT162b2 [148]. Similarly, individuals with previous infection plus vaccination had a lower risk of SARS-CoV-2 reinfection and COVID-19 hospitalization than previously infected people [146]. In another study of 1260 dialysis patients, patients who were not vaccinated had a mortality rate of 24.2%, compared with a mortality rate of 8.6% in patients who experienced breakthrough infections [149]. These data indicate that therapeutic COVID-19 vaccines may become an effective option in the treatment of SARS-CoV-2 infection and the associated disease.

## Therapeutic vaccines against noncommunicable diseases

Cancers were defined as a chronic disease due to their turning into controllable conditions [150]. Therapeutic cancer vaccines have been envisioned as effective tool of cancer immunotherapy [151]. Despite the immense efforts, however, therapeutic cancer vaccines have shown modest benefit in mediating anti-tumor activity in humans. A deeper understanding of the tumor-associated antigens, neoantigens and checkpoint inhibitors has facilitated the improvement of therapeutic cancer vaccines. Meanwhile, vaccines have been developed as therapies against other diseases such as hypertension, dyslipidemia, Alzheimer’s disease, and amyotrophic lateral sclerosis. Based on the promising results in preclinical and clinical trials, vaccines may be an alternative strategy for lifestyle diseases. Here, we describe the representative knowledge and research progress of therapeutic vaccines for cancer, hypertension, Alzheimer’s disease, amyotrophic lateral sclerosis (ALS), diabetes, and dyslipidemia (Table 2).

**Table 2:** Selected preclinical or clinical trials of therapeutic vaccines against chronic non-communicable diseases

Therapeutic vaccines for cancers					
Vaccine type	Vaccine Formulation	Combination agents	Condition	Identifier/ reference	Phase/status
Personalized peptide vaccine	NeoVax (neoantigen + Poly-ICLC)	Ipilimumab	Kidney Cancer	NCT02950766	Phase I Recruiting
	NEO-PV-01 (neoantigen) + Poly-ICLC	Nivolumab	Melanoma, lung, or bladder cancer	NCT02897765	Phase Ib Completed
	GRT-C903 (shared neoantigen prime)/GRT-R904(shared neoantigen boost)	Nivolumab/ipilimumab	NSCLC, colorectal cancer, pancreatic cancer, shared neoantigen-positive solid tumors	NCT03953235	Phase I/II Recruiting
	GRT-C901(patient-specific neoantigen prime)/GRT-R902(patient-specific neoantigen boost)	Nivolumab/ ipilimumab	NSCLC, colorectal cancer, gastroesophageal, adenocarcinoma, urothelial carcinoma,	NCT03639714	Phase I/II Active, not recruiting
	Multiple candidate tumor-derived neoantigens	Pembrolizumab	Advanced Cancer	NCT03568058	Phase Ib Active, not recruiting
	GEN-009 (neoantigen) + Poly-ICLC	Nivolumab/ Pembrolizumab	Cutaneous melanoma,NSCLC, SCC ofhead and neck, urothelial carcinoma, RCC	NCT03633110	Phase I/IIa Completed
	PGV001 (patient specific long peptides+ helper peptides) + Poly-ICLC	Atezolizumab	Urothelial/bladder cancer	NCT03359239	Phase I Completed
	NeoVax(peptides) plus Montanide	Nivolumab/Ipilimumab	Melanoma	NCT03929029	Phase Ib Recruiting
	personalized peptide	Pembrolizumab +imiquimod	Pancreatic and colorectal cancer (advanced)	NCT02600949	Phase I Recruiting
	PGV001(patient specific long peptides+ helper peptides) + Poly-ICLC	Lenalidomide	Solid Tumors	NCT02721043	Phase I Completed
	CDX-1401(DEC-205/NY-ESO-1 Fusion Protein) + Poly-ICLC	Recombinant Flt3 Ligand	Stage IIB-IV melanoma	NCT02129075	Phase II Completed
mRNA-based neoantigen vaccine	RO7198457(mRNA-based)	Atezolizumab	Locally advanced or metastatic tumor	NCT03289962	Phase Ia/Ib Active, not recruiting
	mRNA-4157(lipid encapsulated RNA)	Pembrolizumab	Solid Tumors	NCT03313778	Phase I Recruiting
	mRNA-4157(lipid encapsulated RNA)	Pembrolizumab	Melanoma	NCT03897881	Phase II Active, not recruiting
	RO7198457(mRNA-based)	Atezolizumab+ mFOLFIRINOX	Pancreatic Cancer	NCT04161755	Phase I Active, not recruiting
DNA-based neoantigen vaccine	Neoantigen DNA (TDS-IM system)	Durvalumab	TNBC	NCT03199040	Phase I Active, not recruiting
	GNOS PV02 (Personalized Neoantigen DNA Vaccine)	Pembrolizumab+INO9012(plasmid encoded IL-12)	HCC	NCT04251117	Phase I/IIa Recruiting
**Therapeutic vaccines for hypertension**					
Protein vaccine	PMD3117(Ang I analog with keyhole limpet haemocyanin(KLH))	aluminum hydroxide		[152]	Phase II Completed
Peptide vaccine	CYT006-AngQb (angiotensin II- conjugated to the VLP Qβ)	–	hypertension	[153]	Phase I Completed
		–	Essential Hypertension	NCT00500786	Phase I/II Completed
	ATR12181 (peptide from rat AT1a receptor)	Complete Freund’s adjuvant	Spontaneously hypertension (in rat)	[154]	Preclinic trail
	ATRQβ-001(a peptide derived from human Ang II receptor type 1 conjugated with VLP Qβ)	Aluminum hydroxide	Hypertensive Animals	[155]	Preclinic trail
DNA Vaccine	AGMG0201 (expressing angiotensin II)	–	Mild to moderate essential hypertension	ACTRN12617001192370 [156]	Phase I/IIa Completed
**Therapeutic vaccines for Alzheimer’s disease**					
Peptide vaccine	ABvac40(a conjugate of Aβx-40 with KLH)	Aluminum hydroxide	Mild to moderate Alzheimer’s disease	NCT03113812	Phase I Completed
		Aluminum hydroxide	Mild cognitive impairment Alzheimer Disease	NCT03461276	Phase II Active, not recruiting
	AADvac1 (a synthetic peptide from a tau protein sequence coupled to KLH)	Aluminum hydroxide	Alzheimer’s Disease	NCT02031198	Phase I Completed
		Aluminum hydroxide	Alzheimer’s Disease	NCT02579252	Phase II Completed
	UB311	CpG ODN and alum	Alzheimer’s Disease	NCT00965588	Phase I Completed
		CpG ODN and alum	Mild Alzheimer’s Disease	NCT02551809	Phase II Completed
**Therapeutic vaccines for other diseases**					
Peptide vaccines	tgG-DSE2lim and tgG-DSE5b	Emulsigen-D	Amyotrophic lateral sclerosis (ALS) in mice	[157]	Preclinical trial
	T-cell epitopes within DPP4 conjugated to KLH	Complete/incomplete Freund’s adjuvant	Type 2 diabetes mellitus in mice	[158]	Preclinical trial
	PCSK9Qβ-003(PCSK9 peptide conjugated with VLP Qβ)	Aluminum hydroxide	Dyslipidemia in mice	[159]	Preclinical trial

Source: The clinical trials were from ClinicalTrials.gov or Australian New Zealand Clinical Trials Registry (ANZCTR)

### Neoantigen-based therapeutic cancer vaccines

Successful antitumor immunity requires multiple aspects of tumor immunity, including tumor antigen presentation, T-cell priming and activation, the recognition of tumor cells by T cells, and subsequent effector mechanisms to eliminate tumor cells [160]. Within the tumor microenvironment (TME), pattern recognition receptors of natural killer (NK) cells, neutrophils or macrophages, such as Toll-like receptors (TLRs), recognize pathogen-associated molecular patterns (PAMPs) or damage-associated molecular patterns (DAMPs), activate transcription factors, stimulate cytokine and chemokine production and recruit and activate lymphocytes, finally eliciting innate immunity [161]. Antigen-presenting cells (APCs), such as dendritic cells (DCs), are essential for initiating antitumor adaptive immunity, which captures and recognizes the immunogenic tumor antigens released from dead tumor cells by chemotherapy or immunogenic cell death. This promotes the expression of T-cell costimulatory signals and cytokines by APCs; APC maturation; and antigen uptake, processing and presentation on MHC molecules [162]. These APCs, especially DCs, migrate toward secondary lymphoid organs and interact with naïve CD4^+^ T cells and CD8^+^ T cells, which ultimately results in the priming and activation of T cells [163]. Activated T cells travel back to the TME to induce tumor killing or prevent tumor cell proliferation [16].

Therapeutic cancer vaccines commonly consist of adjuvants and tumor antigens selected with the aim of triggering an innate and adaptive antitumor response against tumor antigens to suppress tumor growth and induce tumor regression. In the early stages of tumor vaccine development, successful therapeutic vaccination against tumors was simply considered to activate tumor-specific T cells and then control tumor growth. Sipuleucel-T, a unique therapeutic cancer vaccine approved by the FDA in 2010, prolonged the overall survival of only 31.7% of male patients with metastatic castration-resistant prostate cancer (NCT00065442) [164]. Moreover, other therapeutic cancer vaccines, such as MAGE-A3 immunotherapeutic (NCT00480025) [165], Belagenpumatucel-L (NCT00676507) [166], tecemotide (L-BLP25) (NCT00409188) [167], and IMA901 (NCT01265901) [168], did not increase overall survival. The mechanisms of immune suppression, resistance and escape mediated by the highly complex heterogeneity of the TME were ignored when these cancer vaccines were designed, which consequently resulted in an inadequate number of T cells, insufficient durability of the T-cell response and failure of T cells to infiltrate the tumor core [169]. Immune checkpoint inhibitors (ICIs), such as anti-CTLA-4, anti-PD1 and anti-PD-L1 antibodies, interfere with the inhibitory pathways of T-cell reactivity, overcoming tumor escape and activating T-cell effector function [170]. The rapidly increasing understanding of immune resistance and escape and the promising efficacy of ICI treatment have encouraged investigators to combine therapeutic cancer vaccines with ICIs. Again, therapeutic cancer vaccines are considered strategies to increase response rates and survival [16].

Effective therapeutic vaccination against tumors depends on the design and screening of a high-quality tumor antigen, efficient antigen uptake by DCs, the sustained activation of CD4^+^ T cells and CD8^+^ T cells, T-cell infiltration into the TME and the strong persistence of the immune response. The success of antigen-specific therapeutic vaccines depends heavily on the choice of antigens in cancer vaccine design. An ideal antigen should be specifically present on the surface of all tumor cells and should be highly immunogenic. For many years, shared tumor-associated antigens (TAAs) have served as the focus of most cancer vaccines. These TAAs are self-molecules abnormally expressed by tumor cells or the “non-self” antigens of oncogenic viruses. They include cancer testis antigens (e.g., MAGE-A1, MAGE-A3, and NY-ESO-1), which are restricted to only immune privileged germline cells and have no or low expression in normal adult somatic cells [171, 172]; differentiation antigens (e.g., tyrosinase, gp100, MART-1, PSA and PAP), which are normally not expressed in adult tissue [173]; overexpressed antigens (e.g., RAGE-1, hTERT, HER2, mesothelin, and MUC-1), which are aberrantly overexpressed in tumor cells compared to normal cells [174]; and oncoviral products (e.g., E6/E7 proteins from HPV) [175]. However, several hurdles are associated with therapeutic vaccination focused on TAAs, resulting in unsuccessful and ineffective antitumor immune responses. This may be due to the low affinity between TAA-specific T cells and antigens due to central and/or peripheral tolerance, the loss of tumor antigen expression, a suppressive TME and collateral damage caused by the expression of some TAAs in nonmalignant tissues [21]. More recently, neoantigens have attracted attention as a subset of nonautologous antigens with individual specificity that are generated by nonsynonymous somatic mutations, frameshifting, insertion/deletion variants, alternative splicing, gene fusions and endogenous retroviruses [176, 177].

#### Neoantigen vaccines

Neoantigens differ from the traditionally used TAAs. Neoantigens are expressed exclusively by malignant cells but lack expression in normal tissues, which prevents collateral damage to nonmalignant tissues. Moreover, neoantigens possess strong immunogenicity toward and high affinity for T cells that are not subject to central tolerance in the thymus [178]. Neoantigen vaccines activate CD4^+^ and CD8^+^ T cells, which directly recognize autologous melanoma cells and discriminate antigens between mutant and wild-type cells [179].

Neoantigens are highly individual-specific and are derived from mutations occurring in the tumor cell genome [176]. Hence, the identification of neoantigens is critical for neoantigen vaccine development. While the tumor mutational burden (TMB) is heavily correlated with neoantigen formation, it is possible that a high level of mutation in the somatic exonic region will lead to increased neoantigen production and then recognition by CD8^+^ T cells [180]. Jaffee and colleagues evaluated the relationship between the TMB and the objective response rate for anti-PD-1/anti-PD-L1 therapy [181], which revealed TMB as a potential biomarker for the response to ICIs [182]. Most tumors can be categorized as having a high TMB, correlating with a correspondingly high number of neoantigens, and are more likely to respond to ICIs. However, a high TMB does not universally indicate the response to ICIs. For instance, a number of melanoma or NSCLC patients with a high TMB do not respond to ICIs, but some patients with renal cell carcinoma (RCC) with a low TMB respond to ICIs [182]. Hence, it is reasonable to assume that mechanisms other than the TMB may contribute to the quality of neoantigens. These mechanisms are as follows [183]: 1) The probability of being a T-cell-recognized neoantigen. A high level of mutation results in the accumulation of neoantigens, but only a small fraction is recognized by T cells. An opportunity for the generation of strong neoantigens exists in patients with a low TMB. 2) The clonality of neoantigens. Neoantigen-specific T-cell responses against clonal mutations have been observed, but not subclonal mutations with the possible loss of neoantigen expression. 3) TCR affinity for neoantigens; 4) Patients’ HLA class I genotype. CHOWELL et al. found that the maximal heterozygosity at HLA-I loci (HLA-A, HLA-B and HLA-C) was strongly associated with extended survival after ICI immunotherapy [184]. Therefore, the accurate prediction of neoantigens or the direct measurement of neoantigens on tumor cells is crucial for clinical success. The rapid development of high-throughput next-generation sequencing technology, including whole-genome sequencing and whole-exon sequencing, has provided opportunities to identify thousands of tumor-associated mutations in individual patients. Additionally, machine-learning-based algorithms for MHC-I (encoded by the HLA-A, HLA-B and HLA-C genes) or MHC II (encoded by the HLA-DR, HLA-DP and HLA-DQ genes) epitope prediction can expedite the identification of immunogenic neoantigens [185, 186]. Therefore, personalized therapeutic cancer vaccine-targeted neoantigens have become a promising method for tumor immunotherapy for individual patients.

Several clinical trials have shown that multiple types of personalized neoantigen vaccines can induce CD4^+^ T cell and CD8^+^ T cell antigen-specific responses and benefit patient survival. A notable study demonstrated that monocyte-derived DCs loaded with personalized neoantigens induced a T-cell-specific immune response in patients with melanoma [187]. Carreno et al. utilized exome sequencing to identify somatic mutations and in silico analysis to assess HLA-A*02:01 peptide-binding affinity, ultimately obtaining neoantigen candidates for each patient with stage III resected cutaneous melanoma. Subsequently, mature DCs loaded with neoantigen candidates were prepared in vitro and then transfused into the patients by intravenous infusion. Most vaccine-induced neoantigen-specific T cells exhibited a type I-skewed phenotype with high amounts of IFN-γ. DCs loaded with personalized neoantigens activated T-cell immunity and enhanced the breadth of antitumor immunity. Three patients benefited from the neoantigen vaccines and showed no autoimmune adverse events.

RNA vaccines are attractive because they carry genetic information for endogenous protein expression, and the neoepitopes can be translated without transcription. RNA vaccines are flexibly manufactured and induce both humoral and cellular immunity [188]. Sahin et al. [189]. first reported an RNA-based polyneoepitope against melanoma (NCT02035956). In this study, candidate neoantigens were identified by comparative exome and RNA sequencing and screened independent of high-affinity binding to HLA class I and II by computer simulation. The prepared RNA vaccine, injected into draining lymph nodes and capable of encoding candidate neoantigens, elicited CD4^+^ T cell and CD8^+^ T-cell responses. Eight of 13 patients who received the RNA vaccine showed no recurrence after 12 months of follow-up.

Similarly, Ott et al. [179]. identified tumor-specific mutations by whole-exome sequencing and RNA sequencing and predicted the binding activity of neoantigens to HLA molecules. The personalized peptide neoantigen vaccines consisting of neoantigens with poly-ICLC adjuvants induced T cell responses, among which 60% of neoantigens activated CD4^+^ T cells and 16% of neoantigens activated CD8^+^ T cells. Four of six vaccinated patients with untreated high-risk melanoma had no recurrence at 25 months after inoculation.

Several types of malignancies, including melanoma, pancreatic cancer, breast cancer and lung cancer, are being treated in clinical trials by neoantigen vaccines [190]. Two clinical trials have demonstrated that personalized neoantigen vaccination was feasible for glioblastoma with a low mutation load, an immunologically ‘cold’ TME and a low response to ICIs [191]. Hilf et al. [192]. prepared two synthesized vaccines: APVAC1 (targeting unmutated antigens) and APVAC2 (targeting neoantigens). Fifteen patients with glioblastomas were successively treated with APVAC1 and APVAC2. In 50 % of patients treated with APVAC1, mainly a CD8+ T-cell response was elicited. A CD4+ T-cell response was induced in 84.7% of patients treated with APVAC2. Vaccination improved the overall survival (OS) and progression-free survival (PFS) to 29.0 and 14.2 months, respectively. Patients in whom glioblastomas were surgically resected received another personalized peptide neoantigen vaccine that elicited neoantigen-specific CD4^+^ and CD8^+^ T-cell responses and showed an accumulation of neoantigen-specific T cells in the tumor site [193].

Neoantigen vaccines based on RNA, DCs or peptides aim to induce CD4^+^ and CD8^+^ T-cell responses and promote T-cell infiltration into the tumor core. The findings from these preliminary clinical trials indicated that neoantigen vaccines could benefit patients with malignant tumors.

#### Combination of neoantigen vaccines with other immunotherapies

**Fig. 4:**
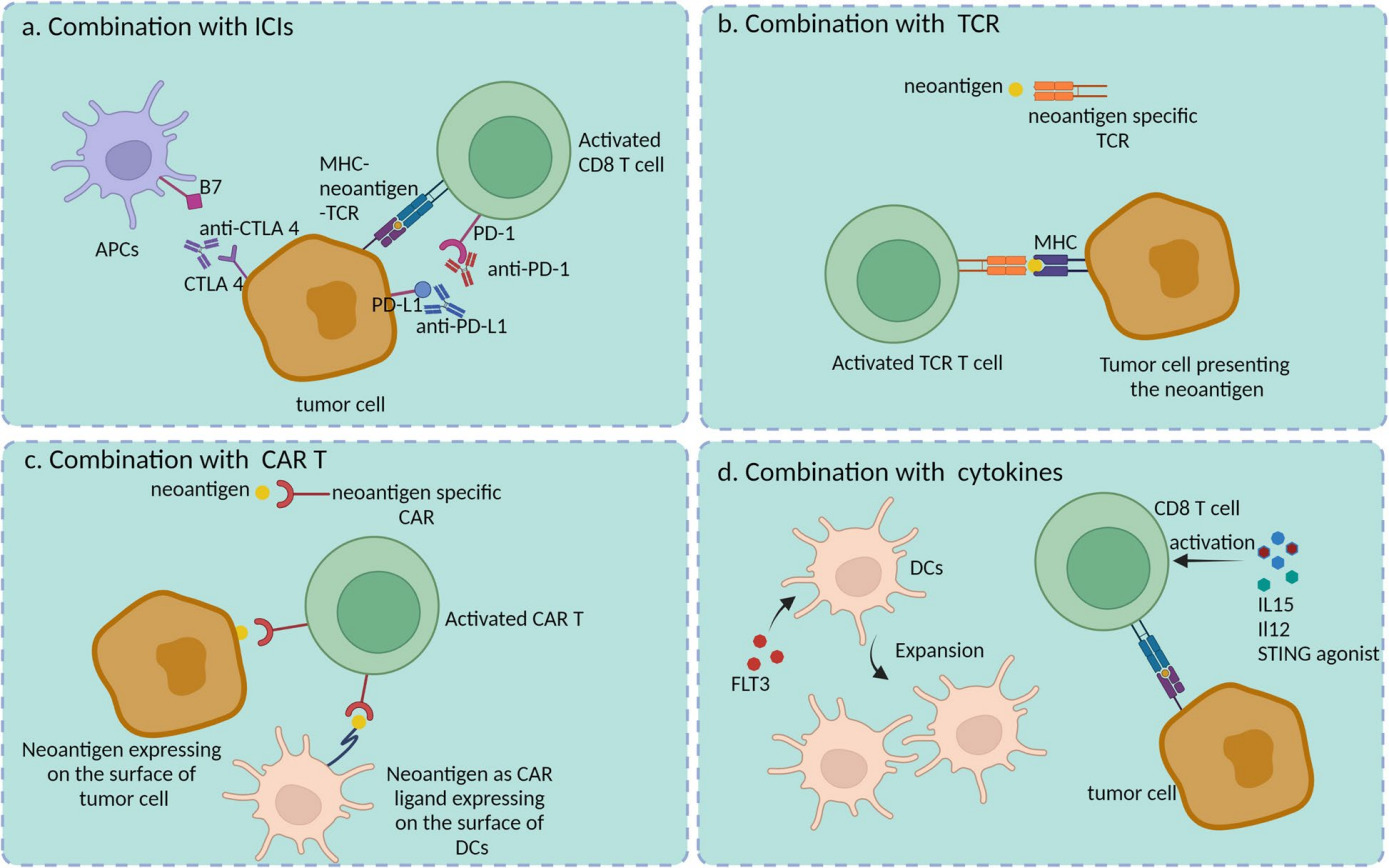
Combination of neoantigen vaccines with other therapied for cancer. **a** Combination of neoantigen vaccines with ICI therapy such as anti-CTLA4, anti-PD-1 and anti-PD-L1 could relieve exhaustion of T cells and APCs; **b** Combination with TCR therapy, T cells were engineered with neoantigens-specific TCR to kill tumor cells; **c** T cells were equipped with neoantigens-specific CAR which could effectively recognize tumor cells which expressed neoantigens. Even neoantigen can be a CAR ligand to be transferred to the surface of DCs, activating the neoantigen-CAR T cells; **d** Cytokines induce the expansion of DCs or enhance the activity of T cells to improve the anti-tumor immunity

Neoantigen vaccines as monotherapy cannot completely eliminate malignant tumors. Several preclinical and clinical trials have investigated the combination of neoantigen vaccines with other immunotherapies, including ICIs, cytokines [194], immune-stimulatory molecules [195, 196], adaptive T-cell therapy, CAR-T therapy, and radiation therapy [197, 198], aiming to activate and expand the antitumor response by reversing the immunosuppressive TME with immune-suppressive cells and the expression of immune checkpoints (Fig. 4) [199].

Tumor vaccines activate the CD4^+^ and CD8^+^ T-cell response, resulting in IFN-γ production [179, 189]. However, IFN-γ regulates the expression of PD-L1 in the TME, inhibiting the efficacy of tumor vaccines [200, 201]. Moreover, several studies have reported that neoantigen-specific T cells express high levels of PD-1 following treatment with monotherapy neoantigen. Yadav et al. [202]. immunized mice with mutated tumor neoantigens to evaluate immunogenicity. They found that the infiltration of neoantigen-reactive CD8^+^ T cells was increased. However, 76.9% of these tumor-specific CD8+ TILs coexpressed PD-1 and TIM-3, which may lead to T-cell exhaustion [203]. Sahin et al. detected the expression of PD-1 on neoepitope-specific T cells and the upregulation of PD-L1 in postvaccination lesions [189]. All evidence indicated that it is rational to combine the neoantigen vaccine with ICIs. Four of six patients with untreated high-risk melanoma benefited from peptide neoantigen vaccines [179]. However, two of six patients (stage IVM1b disease with lung metastases) had disease recurrence evident at follow-up. Both patients were subsequently treated with the anti-PD-1 antibody pembrolizumab for four doses. Complete elimination of tumors was observed in both patients, along with the expansion of neoantigen-specific T cells [179]. Another study also demonstrated that the choice of combination therapy can also significantly affect the therapy outcome [189]. One patient who received the RNA-based poly-neoepitope vaccine showed a strong immune response to neoantigens but experienced multiple relapses due to fast disease progression [189]. This patient was then given the anti-PD-1 antibody pembrolizumab after stopping vaccination. After pembrolizumab treatment, 80% of multiple melanoma lesions were reduced and eventually showed a complete response. Importantly, vaccine-mediated T cells persisted for up to 9 months [189]. Beyond these trials, several clinical trials are currently exploring the efficacy of neoantigen vaccines in combination with ICIs.

The efficacy of vaccines depends on the activated T-cell responses. However, the absence of active T cells in the system or low T-cell infiltration in the TME weakens the function of neoantigen vaccines. Therefore, combinations of neoantigen vaccines and T-cell therapy have the potential to be successfully used to achieve antitumor responses. T cells engineered to express a TCR targeting unique tumor-specific antigens were shown to eradicate large established cancers [204]. Liu et al. [205]. identified neoantigens from epithelial ovarian cancer that activated antigen-specific CD4^+^ and/or CD8^+^ T-cell responses by enhancing antigen processing and presentation. Peripheral T cells were engineered to express TCRs from neoepitope-specific T cells and demonstrated neoepitope-specific reactivity. The combination of neoantigens and adapted T cells is a promising strategy for expanding antitumor immunity.

CAR-T therapy has recently achieved inspiring clinical success in the treatment of hematological malignancies, but it has shown limited efficacy in the treatment of solid tumors owing to several problems, such as suboptimal trafficking of engineered T cells to tumors, antigen loss or heterogeneity, and poor fit with the TME. Amphiphile CAR-T ligands were designed by Ma and colleagues to combine therapeutic vaccines with CAR-T cells to alleviate the problem and enhance efficacy [206]. The amphiphile ligands consisted of an albumin binding/membrane-inserting domain as the tail where a CAR ligand was attached. When injected, amphiphile ligands (amph-ligands) associated with free albumin by binding with the albumin binding domain and were then rapidly trafficked to the draining LNs, where they were then transferred to the membrane of APCs with the exposure of the CAR ligand to activate CAR-T cells [206]. Ma and colleagues evaluated the efficacy of the combination of the amph-EGFRvIII vaccine with EGFRvIII-CAR-T cells in mice with gliomas and observed a great increase in CAR-T-cell infiltration, a significant delay in tumor growth and prolonged survival. Similarly, the enhancement of antitumor immunity was observed in B16F10 tumor-bearing mice.

In the TME, cytokines can suppress tumor cell growth by activating T cells and natural killer (NK) cells and have antiproliferative or proapoptotic activity, playing an important role in tumor treatment. The coadministration of FLT3, expanding DC and NK populations [207], with an RNA vaccine encoding antigen enhanced the priming and expansion of antigen-specific CD8^+^ T cells and T-cell infiltration into tumors [208]. Additionally, Lee et al. [194] utilized an IL15 superagonist, PD-L1 blockade and the tumor-targeted immunocytokine NHS-IL12 to improve the antitumor efficacies of neoantigen vaccines. They found that the combination therapy resulted in a significant increase in T-cell infiltration, enhanced expansion of T cells and efficient tumor clearance. Moreover, costimulatory molecules are necessary for APC function and the full activation of T cells. PancVAX, a neoantigen-targeted vaccine, was administered with the STING adjuvant ADU-V16 to activate neoepitope-specific T cells and promote tumor regression [195]. The addition of anti-PD-1 and agonist OX40 antibodies to the vaccine resulted in durable tumor regression and a survival benefit, which was associated with the reduced coexpression of the T-cell exhaustion markers Lag3 and PD-1 by OX40-targeted therapy [195].

### Therapeutic vaccines for hypertension

Hypertension is the leading cause of cardiovascular disease and premature death worldwide [209]. In 2010, it was estimated that 31.1% of adults (1.39 billion) worldwide had hypertension, with a higher prevalence in low- and middle-income countries [209]. In China, the hypertension disease burden is increasing [210]. Based on the basic principle of vaccination, researchers began to develop therapeutic vaccines to control hypertension, such as ATR12181, pHAV-4Ang IIs, CYT006-AngQb, AngI-R, PMD3117 and ATRQβ-001 [211]. Downham et al. designed a vaccine, named “PMD-3117”, targeting angiotensin I, that is composed of an analog of angiotensin I fused to KLH and aluminum hydroxide adjuvant. PMD-3117 induced a significant immune response both in rats and in humans [212]. Then, the sustained immune response to angiotensin I induction and the efficacy of the antibodies to block the renin system by PMD-3117 were evaluated in a phase II clinical trial [152]. PMD-3117 induced anti-angiotensin I antibodies but did not sufficiently influence blood pressure. However, PMD-3117 significantly reduced the decrease in plasma renin [152]. AngQb is a conjugate angiotensin II vaccine composed of angiotensin II chemically conjugated to virus-like particles derived from the coat protein of the bacteriophage Qb [213]. In a phase IIa study in patients with mild to moderate hypertension, a 300 μg dose reduced blood pressure during the daytime, especially in the early morning (NCT00500786) [213]. All volunteers produced high IgG titers against angiotensin II. Similarly, the peptide vaccines ATR12181 and ATRQβ-001 targeting angiotensin II type 1 receptor showed promising immune responses in a mouse model [214]. Recently, the safety and tolerability AGMG0201(a modified angiotensin II DNA vaccine) were assessed in a phase I/IIa study which enrolled 12 patients with mild to moderate essential hypertension (ACTRN12617001192370) [156]. Patients who received the AGMG0201 immunization elicited anti-angiotensin II antibody, especially in the high-dose group.

### Therapeutic vaccines for Alzheimer’s disease

Alzheimer’s disease (AD) is the most prevalent form of dementia, affecting 50 million people worldwide [215]. In the clinic, small-molecule drugs (such as rivastigmine, galantamine, and donepezil for inhibiting cholinesterase and memantine targeting the NMDA receptor) can relieve symptoms but cannot alter disease progression [216]. Therapeutic vaccines aim to clear the pathogenic peptide and protein aggregation associated with AD, and promise has been demonstrated in slowing cognitive decline in AD patients. Amyloid β-peptide (Aβ) aggregation pathologically results in β-amyloid plaques and cerebral amyloid angiopathy (CAA) deposits [217]. Hyperphosphorylated tau aggregates form neurofibrillary tangles [218]. Therefore, Aβ and tau are targets for candidate therapeutic vaccines against AD. The peptide vaccine ABvac40 is designed to target the C-terminal end of the Aβ_40_ peptide, which is an abnormal mutant of Aβ, and consists of Aβ_33–40_ conjugated with the carrier protein keyhole limpet hemocyanin (KLH) [219]. In a phase I clinical trial, 11 of 12 (92%) patients aged 50 to 85 years with mild to moderate AD received ABvac40 and produced specific anti-Aβ_40_ antibodies (NCT03113812). No ABvac40-immunized patients showed vasogenic edema, sulcal effusion or microhaemorrhages [219]. A phase II clinical trial is currently ongoing to determine the safety, tolerability and immune response of ABvac40 (NCT03461276). AADVvac1 is another peptide vaccine and contains tau_294–395_ coupled to the KLH carrier [220]. A significant protective humoral immune response and reduction in AD-type hyperphosphorylation of tau were detected in tau transgenic rats after AADVvac1 injection [220]. In a phase I clinical trial, 29 of 30 patients who received AADvac1 produced IgG titers and did not experience meningoencephalitis or vasogenic edema (NCT02031198) [221]. The safety and efficacy study of AADvac1 in patients with mild AD in phase II clinical trials was completed in 2019 (NCT02579252). However, the detailed results have not been reported. UB-311 is a novel synthetic peptide vaccine which mainly targets Th2-biased immunity by fusing two synthetic Aβ_1–14_–targeting B-cell peptides with different helper T-cell peptide epitopes [222]. UB-311 elicited high level of antibody targeting Aβ_1–14_ epitopes with 100% responder rate in patients with mild Alzheimer’s disease in the phase I trial (NCT00965588) [222]. UB-311 has completed the phase-II trial in early-to-mild Alzheimer’s disease patients (NCT02551809).

### Therapeutic vaccines for other diseases

Overall, there are some other therapeutic vaccines against chronic diseases, such as amyotrophic lateral sclerosis (ALS), diabetes, and dyslipidemia. Superoxide dismutase 1 (SOD1) misfolding due to mutation is a risk factor for ALS pathogenesis and progression. Zhao et al. designed two peptide vaccines, tgG-DSE2lim and tgG-DSE5b, both targeting SOD1 mutations, to treat ALS [157]. Both vaccines elicited a rapid and sustained Th2-biased immune response and significantly prolonged the survival of hSOD1^G37R^ transgenic mice. Importantly, tgG-DSE5b significantly delayed disease occurrence and progression [157]. The enzyme dipeptidyl peptidase 4 (DPP4) can rapidly degrade glucagon-like peptide 1 (GLP-1), which increases insulin secretion and improves insulin sensitivity, making it a promising therapeutic target for type 2 diabetes [223]. Pang et al. constructed a polypeptide vaccine targeting DPP4 containing the three epitopes within DPP4 conjugated to KLH [158]. DPP4 vaccination of C57BL/6 J mice successfully increased the DPP4-specific titer, inhibited plasma DPP4 activity, and increased the level of GLP-1, which was associated with an increase in both plasma insulin and pancreatic insulin content [158]. Recently, Zhang et al. prepared a proinflammatory cytokine IL-1β-targeted therapeutic vaccine composed of an IL-1β epitope peptide to treat type 2 diabetes [224]. The vaccine improved glucose tolerance and insulin sensitivity and enhanced B-cell function [224]. High levels of low-density lipoprotein cholesterol (LDL-C) lead to an increased risk of atherosclerosis and ischemic cardiovascular diseases. Proprotein convertase subtilisin/kexin type 9 (PCSK9) is a hepatic enzymatic protein that negatively regulates low-density lipoprotein receptor (LDLR), which results in the accumulation of LDL-C [159]. The PCSK9Qβ-003 vaccine, consisting of PCSK9 peptide conjugated with Qβ VLP, significantly elicited PCSK9-specific antibodies and obviously decreased plasma PCSK9 levels, which resulted in a reduction in plasma total cholesterol [159].

## Discussion

Therapeutic vaccines offer a promising and attractive immunotherapy to combat established infectious diseases and chronic noncommunicable diseases by reason of their safety, specificity, effectiveness and even long-lasting response. Unfortunately, most of therapeutic vaccines were at early clinical stage. However, growing understanding of the spatial and temporal immune response elicited by vaccines and novel immunomodulatory approaches would accelerate the clinical feasibility and efficacy of therapeutic vaccines. Considerable efforts on the progress of therapeutic vaccines should be made on three areas: identification and selection of antigens; the choice of antigen delivery; the development of combination therapy. Better knowledge of the pathogenesis contributes to the selection of antigens owing the stable expression and high immunogenicity. For example, the better understanding of the breadth of TAAs, neo-antigens and the native immune response has facilitated and improved the rational vaccine design [16]. The quality of neoepitopes should be further enhanced for the best effect. The adjuvant used and the administration route acts as key determinants of antigen delivery [16]. Each type of therapeutic vaccines own advantages and defects respectively. Protein/peptide vaccines has the easy production, but low immunogenicity. Vector-based vaccines tend to deliver antigens with a high efficacy, but cause safety concerns. DNA vaccines lack high immune response [76]. The addition of adjuvants could effectively stimulate innate immunity and enhance the effect of Protein/peptide vaccines and DNA vaccines. Furthermore, novel carriers would improve the development of protein/peptide vaccines platforms, such as lipoplexes, liposomes and the self-assembling nanoparticles [16]. Additionally, combining with complementary therapies, such as ICIs, CAR-T and TCR-T therapies, will be important to the improvement of therapeutic vaccines. The better effectiveness was observed in the combination of therapeutic neoantigen vaccines with other these immunotherapies. In chronic viral infection, virus-specific T cells gradually become exhausted [225]. Blockade of PD-1/PD-L1 pathway in vivo provided a beneficial effect on therapeutic HBV vaccine in the LCMV mouse model [226].

Moving forward with those issues settled, therapeutic vaccines could be the alternative strategies and even provide a platform for combination therapy against established infectious diseases and chronic noncommunicable diseases, with minimal toxicity and a good safety profile contributing to improving human health.

## Data Availability

Not applicable.
